# The Central Aspects of Pain in the Knee (CAP-Knee) questionnaire; a mixed-methods study of a self-report instrument for assessing central mechanisms in people with knee pain

**DOI:** 10.1016/j.joca.2021.02.562

**Published:** 2021-06

**Authors:** K. Akin-Akinyosoye, R.J.E. James, D.F. McWilliams, B. Millar, R. das Nair, E. Ferguson, D.A. Walsh

**Affiliations:** † Pain Centre Versus Arthritis, University of Nottingham, UK; ‡ Division of Rheumatology, Orthopaedics and Dermatology, School of Medicine, University of Nottingham, UK; § School of Psychology, University of Nottingham, UK; ¦ NIHR Nottingham Biomedical Research Centre, Nottingham University Hospitals NHS Trust, UK; ¶ Institute of Mental Health, University of Nottingham, UK; # Division of Psychiatry and Applied Psychology, School of Medicine, University of Nottingham, UK; †† Rheumatology, Sherwood Forest Hospitals NHS Foundation Trust, Sutton-in-Ashfield, Nottinghamshire, UK

**Keywords:** Osteoarthritis, Central sensitisation, Knee pain, Musculoskeletal, Questionnaire, CAP-Knee, Central Aspects of Pain in the Knee, OA, Osteoarthritis, CS, Central Sensitisation, QST, Quantitative Sensory Testing, MRI, Magnetic Resonance Imaging, PPT, Pressure Pain Detection Thresholds, TS, Temporal Summation, CPM, Conditioned Pain Modulation, IMHW, Investigating Musculoskeletal Health and Wellbeing, DIF, Differential item functioning, CFI, Comparative Fit Index, TFI, Tucker Fit Index, RMSEA, Root Mean Square Error of Approximation, ACR, American College of Rheumatology

## Abstract

**Objectives:**

Pain is the prevailing symptom of knee osteoarthritis. Central sensitisation creates discordance between pain and joint pathology. We previously reported a Central Pain Mechanisms trait derived from eight discrete characteristics: Neuropathic-like pain, Fatigue, Cognitive-impact, Catastrophising, Anxiety, Sleep disturbance, Depression, and Pain distribution. We here validate and show that an 8-item questionnaire, Central Aspects of Pain in the Knee (CAP-Knee) is associated both with sensory- and affective- components of knee pain severity.

**Methods:**

Participants with knee pain were recruited from the Investigating Musculoskeletal Health and Wellbeing study in the East Midlands, UK. CAP-Knee items were refined following cognitive interviews. Psychometric properties were assessed in 250 participants using Rasch-, and factor-analysis, and Cronbach's alpha. Intra-class correlation coefficients tested repeatability. Associations between CAP-Knee and McGill Pain questionnaire pain severity scores were assessed using linear regression.

**Results:**

CAP-Knee targeted the knee pain sample well. Cognitive interviews indicated that participants interpreted CAP-Knee items in diverse ways, which aligned to their intended meanings. Fit to the Rasch model was optimised by rescoring each item, producing a summated score from 0 to 16. Internal consistency was acceptable (Cronbach's alpha = 0.74) and test–retest reliability was excellent (ICC_2,1_ = 0.91). Each CAP-Knee item contributed uniquely to one discrete ‘Central Mechanisms trait’ factor. High CAP-Knee scores associated with worse overall knee pain intensity, and with each of sensory- and affective- McGill Pain Questionnaire scores.

**Conclusion:**

CAP-Knee is a simple and valid self-report questionnaire, which measures a single ‘Central Mechanisms’ trait, and may help identify and target centrally-acting treatments aiming to reduce the burden of knee pain.

## Introduction

In individuals with knee osteoarthritis (OA), joint pathology only weakly predicts the pain severity^1^, and up to 20% of people with severe OA report unsatisfactory pain relief following arthroplasty that replaces the affected joint^2^. Central pain mechanisms act through the central nervous system to augment knee pain severity^3^, and predict poor outcome following peripherally targeted treatments such as arthroplasty^4^. Central sensitisation (CS) is one such central mechanism, defined as ‘increased responsiveness of nociceptive neurons in the central nervous system to their normal or subthreshold afferent input’^5^. Pain is a sensory and emotional experience, and not only a neuronal response. Therefore central factors other than nociceptive neuronal activity might augment pain, including changes in brain connectivity or appraisal. The extent to which central mechanisms augment knee pain varies between individuals. Treatments that target central pain mechanisms can reduce knee pain severity^6^, and might improve response to peripherally targeted treatments^7^.

Quantitative sensory testing (QST) or magnetic resonance imaging (MRI) approaches assess complex pathophysiological central mechanisms such as CS or other higher brain mechanisms linked to central pain mechanisms^5^. Self-report questionnaires also assess manifestations linked to central mechanisms (including psychological and somatic disturbances), and are less resource-intensive and feasible during normal clinical encounters. However, a comprehensive, validated mechanism-based self-report questionnaire is not currently available to identify people with knee pain that is importantly augmented by central pain mechanisms.

Responses to questionnaires addressing individual characteristics of anxiety, depression, catastrophizing, cognitive-impact, sleep, fatigue, pain distribution and neuropathic-like pain have each been associated with experimental markers of CS (including QST and neuroimaging biomarkers), and with pain outcomes in people with knee pain^2^^,^^8^, ^9^, ^10^, ^11^, ^12^, or some other chronically painful conditions^7^^,^^13^. We previously selected 8 self-report items, each representing one of these eight characteristics based on expert opinion (at least moderate agreement between experts on relevance to central pain mechanisms in knee OA), strength of association with the scales measuring the component characteristic, and with Pressure Pain detection Threshold (PPT) at a site distal to the affected knee (taken as an indicator of CS)^9^. We showed by factor analytic approaches, that these eight items contributed to a single underlying trait^9^. This ‘Central Mechanisms’ trait was associated with PPT at a distal site, and with knee pain severity more strongly than was any one of the eight characteristics alone^9^, and was a better predictor of future knee pain than other clinical predictors^8^. Existing questionnaires, including the short-form Central Sensitization Inventory and the Generalized Pain Questionnaire which were originally designed to assess central sensitivity syndromes such as fibromyalgia^14^^,^^15^ omit important characteristics that have been associated with QST-evidence of central pain mechanisms in people with knee pain. These include neuropathic-like pain qualities and cognitive-impact^9^^,^^12^^,^^16^, ^17^, ^18^, ^19^.

Clinical decision support tools have potential to greatly enhance a clinician's ability to carefully subgroup patients, and inform mechanism-based treatment allocation in primary care settings. Employing such tools could inform choice of tailored stratified treatment pathways for individuals with knee OA pain. However, such tools are not widely available for widespread use in clinical practice.

We therefore have developed a simple questionnaire (Central Aspects of Pain in the Knee; CAP-Knee) comprising self-report items measuring each of the eight characteristics which contribute to the Central Mechanisms trait in people with knee pain. We hypothesised that this Central Mechanism trait is associated with both sensory and emotional components of knee pain. We show that CAP-Knee is interpreted appropriately, psychometrically valid and reliable, and associated with sensory and affective components of pain severity in people with knee pain.

## Methods

### Study design

A mixed methods, cross-sectional observational study combining thematic analysis of interviews with people with knee pain, with quantitative analysis of questionnaire psychometric properties and external validity.

### Participants and recruitment

**Fig. 1:**
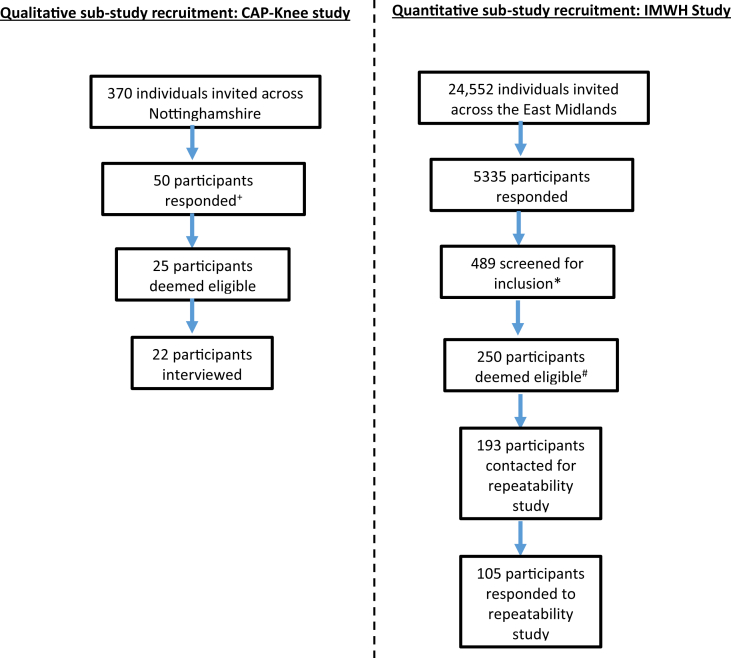
Recruitment flowchart for the quantitative and qualitative assessment sub-studies.+39 out of 50 individuals responding to study between December 2017 and June 2018 consented to further contact and were therefore screened for study eligibility. ∗489 people who had provided baseline questionnaire data to the IMHW study between May 2018 and December 2018 were screened for inclusion, of whom 250, each registered with one of 32 different general practices within the East Midlands region, met the eligibility criteria for this study. ^#^Data from the first 250 participants who completed the CAP-Knee within the IMW&H study and met the inclusion criteria for psychometric assessment of the CAP-Knee were assessed. To test repeatability, 193 of the 250 participants were mailed within 7-days of receipt of their questionnaires with an invitation to complete the CAP-Knee questionnaire a second time. 105 of the 193 participants completed and returned the CAP-Knee questionnaire a second time.

Eligible participants were aged ≥40 years, reported knee pain on most days for the past month, provided informed consent, and could communicate in English. Participants were excluded if they reported having an inflammatory arthritis such as Rheumatoid Arthritis. Details of participant recruitment are provided in Fig. 1.

Small samples (5–15 participants) may fail to uncover common problems in questionnaire evaluation studies, thus a default sample size of 30 participants is endorsed for cognitive interviews^20^, ^21^, ^22^. Recruitment ended when data saturation had been reached, and the research team confirmed when no new themes emerged.

All participants contributing to Rasch or repeatability analysis reported that their knee was their most bothersome joint pain, and that their knee pain had lasted ≥4 weeks. Participants were recruited from those consenting to further contact in the Investigating Musculoskeletal Health and Wellbeing (IMH&W) study (clinicaltrials.gov NCT03696134)^23^***.*** For Rasch analysis, data from 250 to 500 participants may provide accurate and stable person and item estimates, as well as a good balance for statistical interpretation of the fit statistics^24^. Additionally, it is recommended that a minimum sample size for repeatability studies should be 50^25^. Thus, for the Rasch study and the nested repeatability study, complete data for 250 participants were included.

IMH&W participants were recruited from GP surgeries, or a database of participants in previous studies undertaken by the University of Nottingham who had consented to be contacted with information about future research. Ethical approval for interviews was obtained from the Nottingham Research Ethics Committee 2 (17/EM/0480), and for nested recruitment and questionnaire analysis from the London Central Research Ethics Committee (18/LO/0870).

### Self-report questionnaire assessment

**Fig. 2:**
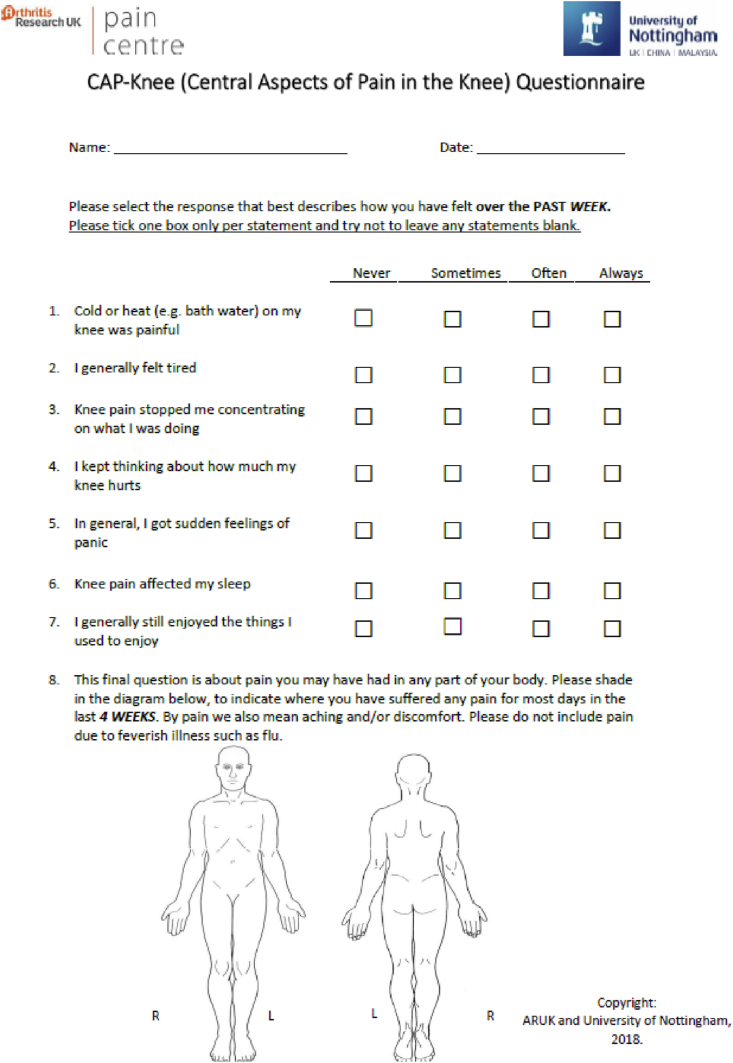
Recruitment flowchart for the quantitative and qualitative assessment sub-studies.+39 out of 50 individuals responding to study between December 2017 and June 2018 consented to further contact and were therefore screened for study eligibility. *489 people who had provided baseline questionnaire data to the IMHW study between May 2018 and December 2018 were screened for inclusion, of whom 250, each registered with one of 32 different general practices within the East Midlands region, met the eligibility criteria for this study. ^#^Data from the first 250 participants who completed the CAP-Knee within the IMW&H study and met the inclusion criteria for psychometric assessment of the CAP-Knee were assessed. To test repeatability, 193 of the 250 participants were mailed within 7-days of receipt of their questionnaires with an invitation to complete the CAP-Knee questionnaire a second time. 105 of the 193 participants completed and returned the CAP-Knee questionnaire a second time.

Data for age, sex, weight and height, physician-diagnosed conditions, CAP-Knee questionnaire, and question groups 1–20 from the McGill Pain Questionnaire^26^ were collected by self-report from all participants. Weight and height were used to calculate body mass index (BMI)^27^. Participants were asked to state the joint with the most bothersome pain, and to rate its severity on an 11-point Likert scale (‘over the past 4 weeks, how intense was your average pain or the average aching feeling in your most bothersome joint, where 0 is ‘no pain’ and 10 is ‘pain as bad as could be’?). In the current study, items originally selected from a larger item pool used in the Knee Pain in the Community study^28^ were reworded and response options modified to provide a consistent structure and response format across the newly developed questionnaire. The final version of the CAP-Knee following item refinement is shown in Fig. 2.

The CAP-Knee questionnaire comprises eight items which assess each of the eight characteristics linked to Central Mechanisms of knee pain. Each of the items originated from established questionnaires measuring Neuropathic-like pain^29^, Fatigue^30^, Cognitive impact^31^, Catastrophizing^32^, Anxiety and Depression^33^, Sleep disturbance^34^, and Pain distribution^35^.

### Cognitive interviews

KAA conducted all cognitive interviews at the University of Nottingham. Eligibility was confirmed by asking participants “Have you had knee pain on most days of the last month?”, and knee OA clinical classification used American College of Rheumatology (ACR) criteria^36^. Participants completed the CAP-Knee questionnaire immediately before the interview.

Interviews used probing techniques that were standardised according to an interview guide (Supplement 1). To elicit difficulties or comments related to interpretation of questionnaire items^37^, probes were developed for each of the four-stage question response model, including stages of comprehension, retrieval, judgement and response formulation^38^. Interviews were audio-recorded, transcribed verbatim using a transcription service, and transcription accuracy was checked against recordings.

The full text of each transcript was coded and analysed with the aid of the NVivo 12 qualitative software programme^39^. Transcripts were iteratively analysed in groups of five or six until data saturation was achieved. Throughout qualitative analyses, disagreements or questions were discussed and interpretations were validated within the research team (KAA, DAW, RdN, EF), to minimise the influence of researcher subjectivity and preconceptions on identifying potential themes^40^. Following assessment of the original version of the CAP-Knee (Supplementary Fig. 1), a further wave of five cognitive interviews after revision of CAP-Knee items was undertaken using the above procedures.

Content analysis adopted a summative approach seeking to assign codes and sub-codes across participant responses within each transcript, following Tourangeau's question response model^37^. In order to guide item revision, problems were identified based on sub-codes of each main code of comprehension (completely-, partially or not aligned to the researchers' intended meaning of each item), retrieval (no-, partial- and complete-retrieval difficulty), judgement (certain initial or uncertain initial response) and response formulation (consistent or inconsistent) - supplement 2. One researcher (KAA) categorised each participant based on quotes contributing to sub-codes across each item^41^. An item was revised if (i) emerging themes were not aligned with researcher's interpretation, and (ii) the item was not well understood by a majority of participants (including complete non-alignment, complete retrieval difficulty, uncertain initial response and no response consistency)^42^.

Thematic analyses also informed item revision by assessing the range of interpretations provided by participants for each CAP-Knee item. An inductive approach to thematic analysis was used to identify themes that were exhaustive and mutually exclusive. Analysis of the transcripts followed a sequential process^43^ allowing the researcher to search for themes and patterns across the entire dataset, rather than within participant interviews. Themes were extracted according to DeSantis and Ugarriza^44^. Identified themes were reduced or expanded to account for various interpretations, and meaning was attributed to the interpretation and experiences expressed by participants about each item^45^.

### Statistical analysis

Based on our previous study^9^ and prior to Rasch analysis, CAP-Knee items one to seven were scored 0 to three according to responses ‘never’, ‘sometimes’, ‘often’, or ‘always’. Item eight was allocated a binary score of ‘0′ or ‘3’ based on pain reported at one knee, plus any other additional region below the waist, by shading on the pain distribution manikin^9^. All subsequent analyses used Rasch transformed scores according to findings made in the current study.

### Rasch analysis

Rasch analysis was performed as described in a previous study^46^, using the Test Analysis Modules (TAM) package within the R programmer software^47^. *Differential item functioning (*DIF) was explored for sex (males and females) and age (<64 years, 64–71 years and over 71 years), using the Mantel–Haenszel test. Residual Mean Square (MNSQ) values between 0.7 and 1.3 were deemed acceptable fit residuals for each item response category^48^. Where disordered response thresholds were observed, items were re-scored by collapsing appropriate adjacent response options^49^^,^^50^. The person separation index (PSI) was calculated to estimate measurement reliability of CAP-Knee, with PSI= >0.70 set as the cut-off for reliability^51^^,^^52^.

### Confirmatory factor analysis (CFA) and floor/ceiling effects

To determine whether CAP-Knee data fit a one-factor model, CFA within a structural Equation Modelling (SEM) framework used M*Plus* version 7.4^53^ as previously described^9^. Floor and ceiling effects were considered present if >15% of respondents achieved the lowest/highest possible scores (50).

### Reliability and associations with knee pain

Reliability was assessed using Stata 14.2^54^. Item-redundancy and internal-consistency were investigated by calculating Cronbach's alpha (α), with values < 0.70 indicating poor internal consistency, and values >0.90 indicating item redundancy^55^. Test–retest reliability was assessed by Intra-class Correlation Coefficient (ICC_2, 1_)^56^.

MPQ responses were summed to give a single score for knee pain intensity, and summated ranks of descriptive terms within groups 1–10 and 11–15 were used to derive sensory and affective pain subscales respectively^26^. Associations between CAP-Knee scores and MPQ total scores for knee pain severity were calculated using Pearson correlation coefficients, and between CAP-Knee and McGill Pain Questionnaire scores using Spearmans rho. Multivariable linear regressions were used for associations between CAP-Knee and pain scales, and were adjusted for age, BMI and sex.

## Results

### CAP-knee items interpretation

Twenty-two cognitive interviews were conducted (participant characteristics in Table I), comprising four transcript groups, at which point data saturation was achieved. Mean interview duration was 29 min (range 16–57 min). Twenty-one participants (95%) fulfilled ACR clinical classification criteria for knee OA, of whom 11 (52%) had bilateral knee OA. Participant interpretation of all items in the final CAP-Knee questionnaire aligned well with their intended meanings (Table II), although participants interpreted each item according to multiple constructs. Table III provides example quotes for each theme and sub-theme.

**Table I:** Characteristics of study participants

Characteristic (units or possible score range)	Total study population (*n* = 250)	Knee most painful joint (*n* = 169)	Reliability subgroup (*n* = 76)	Interviews (*n* = 22)∗
Age (y)	71 (64–77)	71 (64–77)	71 (66–78)	66 (59–74)
Female sex	158 (63%)	96 (58%)	49 (72%)	15 (68%)
BMI (kg.m^−2^)	28 (25–32)	27 (24–32)	28 (24–32)	30 (27–35)
CAP-Knee (0–16)†	7 (5–10)	7 (5–10)	7 (5–9)	6 (4–8)
Joint pain (0–10)	6 (4–8)	6 (4–7)	5 (4–7)	6 (4–7)
McGill total (0–78)	16 (9–25)	16 (8–25)	14 (9–22)	10 (6–11)
McGill affective (0–14)	1 (0–3)	1 (0–2)	0 (0–2)	0 (0–0)
McGill sensory (0–42)	11 (6–16)	11 (6–15)	10 (7–13)	7 (4–11)

Data are median and interquartile range (IQR) or n (%).

∗ Following examination of both knees across participants, 21 (95%) fulfilled the ACR clinical classification criteria for knee OA at any joint, of which 10 (48%) had unilateral OA, and 11/21 (50%) had bilateral OA.

† Rasch transformed scored.

**Table II:** Themes and subthemes identified for each item included within the CAP-Knee Scale

Item	Participants with responses related to codes of poor item function	Key theme (number of participants contributing to theme)	Subtheme (number of participants contributing to subtheme)
1. Neuropathic-like pain (‘Cold or heat touching my knee was painful’)∗	10 (59%)	Thermal allodynia (*n* = 12)	–
		Weather induced pain (*n* = 9)†	
		Thermotherapy (*n* = 5)†	
Item one revised: Neuropathic-like pain (‘Cold or heat (e.g., bath water) on my knee was painful ‘)∗	1 (20%)	Thermal allodynia (*n* = 5)	–
2. Fatigue (“I generally felt tired”)	5 (23%)	Source of fatigue (*n* = 17)	Physical exertion (*n* = 13)
			Sleep disturbance (*n* = 5)
			Other fatigue sources (*n* = 7)
		Fatigue relief (*n* = 8)	–
3. Cognitive-impact (“Knee pain stopped me concentrating on what I was doing”)	7 (32%)	Task distraction (*n* = 10)	–
		Hypervigilance (*n* = 12)	
4. Catastrophizing (“I kept thinking about how much my knee hurts”)	1 (5%)	Causes and consequences (*n* = 11)	–
		Avoidance behaviours (*n* = 9)	
5. Anxiety (“In general, I got sudden feelings of panic”)	0 (0%)	Fear (*n* = 15)	Fear of what happens in the knee (*n* = 7)
			Fear of falling over (*n* = 6)
			Fear for the future (*n* = 3)
6. Sleep (“Knee pain affected my sleep”)	2 (9%)	Sleep disturbance (*n* = 21)	Knee pain interrupting sleep (*n* = 16)

			Other painful sites disturbing sleep (*n* = 7)
		Use of sleeping aids (*n* = 8)	–
7. Depression (“I generally still enjoyed the things I used to enjoy”)	2 (9%)	Social function (*n* = 11)	–
		Physical limitation (*n* = 14)	
8. Pain Distribution (“The final question is about ‘pain that you may have had in any part of your body, please shade in the diagram below to indicate where you have suffered any pain for most days in the last 4 weeks. And by pain, we mean aching and discomfort, but we don't mean pain due to feverish illnesses such as flu.“)	0 (0%)	Painful sites (*n* = 17)	Nature of pain (*n* = 14)
			Impact of pain (*n* = 5)
			Help-seeking experiences (*n* = 9)


∗ All themes emerged from discussions across all participants (*n* = 22), except for the neuropathic-like pain item where the original item in the developmental questionnaire was used in the first three rounds of interviews (*n* = 17), and the revised item used in the final (4^th^) round of interviews (*n* = 5).

† - themes not aligned with intended meaning of the item by the researchers.

**Table III:** Participant quotes supporting themes and subthemes identified for each item included within the CAP-Knee Scale

Item	Key theme (*Participant Quote*)	Subtheme (*Participant Quote*)
1. Neuropathic-like pain	**Thermal allodynia** (“*Well if I put something too cold on it, it really seizes the knee*.” –Interview 1)	–
	**Weather induced pain** (*“So you know when it's been winter and the cold from the winter makes my leg ache worse!”* – Interview 10)	
	**Thermotherapy** (*”… because I think hot water sometimes will ease it [knee pain] but it doesn't at times.” –*Interview 19)	
2. Fatigue (“I generally felt tired”)	**Source of fatigue**	**Physical exertion** (*”… in the past week yes, I have because I've been, I had a lot of making stimulation doing work and it's made me feel tired.” -* Interview 3)
		**Sleep disturbance** (“*Sometimes the pain of it just keeps me awake, that's why it makes me tired*.” – Interview 20″)
		**Other fatigue sources** (“*But I'm 82. I've also got a thyroid problem. And that can make me tired. So it's a combination thing, I'm afraid*.” – Interview 4)
	**Fatigue relief** (“*Well sometimes it tires you out. If it's aching it does make you tired. So, yeah, I just sit down and res*t.” – Interview 9)	–
3. Cognitive-impact (“Knee pain stopped me concentrating on what I was doing”)	**Task distraction** (*“Um, probably say if you're sitting writing or something, would you be able to concentrate if your knee was hurting you? And if you ask me that, I would probably say I would be able to concentrate to begin with and then my knee would niggle away at me. I'd have to stop writing and get up and straighten my legs and then go back to it, yeah.”* – Interview 11)	–
	**Hypervigilance** (*“But if say I wanted to get up and go to the toilet, I have to think about it. You know what I mean? So anything you're doing, it's kind of there.*” – Interview 2)	
4. Catastrophizing (“I kept thinking about how much my knee hurts”)	**Causes and consequences** (”*… I sometimes wonder if that [step exercise] damaged my knee! Because I used to go all the time.*” – Interview 7)	–
	**Avoidance behaviours** (“*Um, well if I'm sitting in, say I'm relaxing, and if I move my leg over it'll start hurting so I'm thinking about, you know, I've got to keep still, you know* …” – Interview 21)	
5. Anxiety (“In general, I got sudden feelings of panic”)	**Fear**	**Fear of what happens in the knee** (“*That the knee's going to pop out. Let's say going upstairs, sometimes it feels as though the bones have gone ‘bip’ and I think is it going to pop out*” – Interview 1)
		**Fear of falling over** (“*I'm so afraid of going, falling, or tripping over something because I can't lift my bloody leg high enough*.” – Interview 16)
		**Fear for the future** (“*Because I think to myself oh if I can't, what am I going to do, if I can't walk, what will I do.”* – Interview 15)
6. Sleep (“Knee pain affected my sleep”)	**Sleep disturbance**	**Knee pain interrupting sleep** (*“Yes it means it's exclusively when I turn over. I'll be fast asleep, and you know, you turn over onto your position, and that's when I feel it. Yeah. So it sort of jolts me awake.”* - Interview 4)

		**Other painful sites disturbing sleep** (“*I wouldn't say it's [knee pain's] woke me up quite like that in the past week, there's been something added to that, that's been part of waking me up, just generally because the pain's across all the body.“–* Interview 6)
	**Use of sleeping aids** (“*You know, I'm afraid I do rely on sleeping pills occasionally if things are bad. You know*.” – Interview 4)	–
7. Depression (“I generally still enjoyed the things I used to enjoy”)	**Social function** (”-*But in life in general, I suppose, [long pause] it's OK, but I just can't join in, you know, with the things that he [husband] likes to do, and whatever, and we used to do together. So* -“- Interview 2)	–
	**Physical limitation** (“*Yeah, well I've not really stopped enjoying them. Yeah, OK, I've been limited to stuff that – I've been just limited, the knee's limited it, but I've still done it.“-* Interview 8)	
8. Pain Distribution (“The final question is about ‘pain that you may have had in any part of your body, please shade in the diagram below to indicate where you have suffered any pain for most days in the last 4 weeks. And by pain, we mean aching and discomfort, but we don't mean pain due to feverish illnesses such as flu.“)	**Painful sites**	**Nature of pain** (“*Well, like, just my left leg, knee there, gives me the actual pain and I say that back bit, it just feels like pinching, like a pinching type of pain.”* – Interview 5)
		**Impact of pain** (“*It was quite restricting at first. I can't get my arm round the back of my head to do my hair and things like that*.” – Interview 11)
		**Help-seeking experiences** (“*But going to the doctors and he said ‘I think you might have fibro-‘, ‘I want a second opinion’, so I waited and waited and then went back, then they noticed I had this Meniscus Tear, so that could have been doing it because it puts strains on other bits, but I went to see a consultant and he said ‘you've got like fourteen points of the fibromyalgia’* …” Interview 10)

Single participant quotes are provide for each theme/subtheme.

Fatigue and sleep items were interpreted as discrete constructs by most participants, although five attributed fatigue to sleep disturbance. Most (*n* = 13) participants described the fatigue item in terms of physical exertion as a source of fatigue. Seven participants attributed fatigue to other causes (older age, medical problems including thyroid problems, diabetes, and fibromyalgia, or medication). Fatigue was also described in terms of a need to rest in order to obtain relief (*n* = 8). Sleep disturbance was related to knee pain or discomfort (*n* = 16) while trying to sleep, but also to the impact of other painful sites (*n* = 7). Participants often described their sleep disturbance by reference to aids that they used to help them sleep (*n* = 8), including pharmacological analgesics or sleeping pills, or non-pharmacological aids such as cushions placed between their knees.

The cognitive item was described in terms of task distraction (*n* = 10) or hypervigilance (*n* = 12). The catastrophizing item was interpreted in terms of causes and consequences of knee pain (*n* = 11), or of avoidance behaviours (*n* = 9).

The anxiety item was related to three subthemes, fear for the integrity of their knee (*n* = 7), fear of falling over (*n* = 6), and fear of future disability (*n* = 3). The depression item was related to aspects of both social (*n* = 11) and physical (*n* = 14) functioning that participants no longer enjoyed.

The pain distribution manikin was variously interpreted by participants, who indicated different qualities or diagnoses for pain at different sites and at different times (*n* = 14), or indicated sites of pain for which they had sought professional help (*n* = 9), or which had different impacts on their life (*n* = 5).

The neuropathic-like pain item in the developmental questionnaire was not well understood by 59% of participants (Table II). Participants referred to weather-induced pain or thermotherapy which were not aligned with the intended meaning of thermal allodynia. The rewritten item in the final CAP-Knee questionnaire (Fig. 1) references an example of tangible physical stimulus: ‘Cold or heat (e.g., bath water) on my knee was painful’, and was understood well by all participants.

### Rasch modelling, scale and item responses

Baseline characteristics for study participants across the interview and Rasch/repeatability studies are shown in Table I. Likelihood ratio test showed significant differences (p< 0.001) between the partial credit formulation and the rating scale model. The partial credit formulation therefore was used, thereby avoiding assumption of uniform threshold distance across all items.

The summary item-fit statistics from analyses of CAP-Knee scores with four response levels per item indicated misfit to the Rasch model, with significant item–trait interaction [X^2^(df) = 63 (28); p< 0.001] (Table IV). The cognitive-impact item showed misfit (Table V). CAP-Knee was well targeted, but the sleep disturbance item exhibited disordering of the step difficulty (i.e., the difficulty of a higher step was lower than that of its adjacent lower step), displaying a disordered response threshold (Supplementary Fig. 2). Principal Components Analysis of the residuals found that all eight items loaded on the first component. Eleven (4% [Binomial CI: 2–8%]) of 246 *t*-tests were significant (Table IV). Four items (neuropathic-like pain, fatigue, anxiety and depression) showed misfit for outfit values in one or more response options (Table V). CAP-Knee item residuals demonstrated no correlations (*r* < 0.3) between items. None of the items exhibited non-uniform DIF for age or sex. None of the items showed uniform-DIF for age, however, the pain distribution item showed uniform-DIF (*P* = 0.03) for sex.

**Table IV:** Summary item–person interaction statistics for CAP-Knee using the partial credit model

Model	Χ^2^ (df)	*P* value	Item fit residual (mean)	Item fit residual (SD)	Person fit residual (mean)	Person fit residual (SD)	PSI	Percentage of significant *t*-tests (95% CI)
Scores not Rasch transformed	63 (28)	<0.05	0.79	1.35	0.01	1.09	0.8	4.43% (2.23–7.79%)
Scores Rasch transformed	52 (28)	<0.05	0.19	1.34	0.02	1.28	0.73	4.43% (2.23–7.79%)
Ideal value	–	>0.05	0	1	0	1	≥0.70	<5%

Rasch transformation comprised collapsing responses ‘Often’ and ‘Always' each scored 2, whereas non-transformed scores were ‘Often’ = 2, ‘Always' = 3. PSI; Person Separation Index. *N* = 250.

**Table V:** Fit statistics for CAP-Knee items

Items	Scores not Rasch transformed				Scores Rasch transformed			
	Difficulty logit	SE logit	Outfit MNSQ	Infit MNSQ	Difficulty logit	SE logit	Outfit MNSQ	Infit MNSQ
1. Neuropathic- like pain	2.03	0.11	0.86	0.91	1.6	0.12	0.86	0.92
2. Fatigue	−0.12	0.08	0.98	0.99	−0.99	0.11	0.94	0.94
3. Cognitive-impact	1.09	0.09	0.59	0.59	0.45	0.10	0.59	0.60
4. Catastrophizing	0.59	0.08	0.72	0.72	−0.09	0.10	0.73	0.75
5. Anxiety	2.39	0.12	0.85	0.94	1.96	0.13	0.88	0.93
6. Sleep disturbance	0.78	0.09	0.70	0.72	0.21	0.10	0.67	0.69
7. Depression	0.11	0.08	1.02	1.07	−0.64	0.10	0.93	1.02
8. Pain distribution	0.17	0.15	1.23	1.16	−0.45	0.16	1.37	1.19

Rasch transformation comprised collapsing responses ‘Often’ and ‘Always' each scored 2, whereas non-transformed scores were ‘Often’ = 2, ‘Always' = 3.. Negative difficulty logits indicate items that are easier to endorse, and positive measures indicate items that are more difficult to endorse. Cognitive-impact and sleep disturbance items displayed misfitting values for infit or outfit (MNSQ outside the range 0.7–1.3). *N* = 250. MNSQ = Mean square residual; SE = Standard Error.

CAP-Knee analysis was modified by collapsing response categories until the thresholds demonstrated sequential levels of severity. Merging the highest categories 3 (*‘Always’*) and 2 (*‘Often’*) for the first seven items produced a scale that performed better than using four response levels (Table IV, Table V, Fig. 1). The final scale, in which each item was allocated a score from 0 to two using collapsed respones (possible total score range; 0 to 16) was unidimensional, with no local item dependency observed, although the pain distribution item still showed uniform-DIF for sex. The Rasch-transformed CAP-Knee scoring regime is shown in Supplement 3.

### Confirmatory factor analysis and reliability

Factor analysis confirmed the one-factor model (comparative fit index = 0.98; Tucker Lewis Index = 0.97; model X^2^(df) = 38 (20); root mean square error of approximation = 0.08). All eight items loaded significantly on to the single latent factor, which we named ‘Central Mechanisms’ (Table VI). Cronbach's alpha was 0.74. Of the respondents, one (0.4%) had the minimum CAP-Knee score of 0, and two (0.8%) had the maximum score of 16. Pairs of complete questionnaires were obtained from each of 76 of 105 participants who completed the CAP-Knee the second time for the reliability study (Table I). The median interval between the first and second assessments was 20 days (IQR 17–24 days). The reliability intra-class correlation coefficient (ICC_2, 1_) was 0.91 (95% CI 0.86 to 0.94).

**Table VI:** Item loading for Rasch-transformed CAP-Knee scores

Characteristic: Item wording	Loading
1. ***Neuropathic-Like pain:*** Cold or heat (e.g., bath water) on my knee was painful	0.559∗∗
2. ***Fatigue:*** I generally felt tired.	0.470∗∗
3. ***Cognitive-impact:*** My knee pain stopped me concentrating on what I was.	0.864∗∗
4. ***Catastrophizing:*** I kept thinking about how much my knee hurts.	0.748∗∗
***5. Anxiety:*** In general, I got sudden feelings of panic.	0.692∗∗
6. ***Sleep disturbance:*** My knee pain affected my sleep.	0.736∗∗
7. ***Depression:*** I generally still enjoyed the things I used to enjoy.	0.450∗∗
8. ***Pain Distribution:*** This final question is about pain you may have had in any part of your body. Please shade in the diagram below, to indicate where you have suffered any pain for most days in the last four WEEKS. By pain we also mean aching and/or discomfort. Please do not include pain due to feverish illness such as flu.	0.158∗

CFA of the Rasch-transformed CAP-Knee scores (responses for items 1–7 collapsed to three levels). **Loading;** factor loading coefficient for item loading to the single ‘Central Mechanisms' factor, *n* = 250, ∗∗p < 0.01, ∗p < 0.05.

### Association of CAP-Knee scores with knee pain severity

We hypothesised that because central mechanisms augment nociceptive pain from the knee, CAP-Knee would be associated with both sensory and emotional components of knee pain in participants who reported knee as their most painful joint (*n* = 169).

Higher CAP-Knee scores were indeed associated with knee pain severity, measured using a 0–11 Likert scale (*r* = 0.62, p < 0.001). Associations between knee pain severity and CAP-Knee scores remained significant in a multivariable linear regression model which included age, sex and BMI as covariates (*B* = 0.33 (95% CI 0.25–0.41), p < 0.001). CAP-Knee was also positively associated with total McGill Pain Questionnaire scores (rho = 0.61 (95% CI 0.59–0.64), p < 0.001), as well as with each subscale for Affective or Sensory Pain (rho = 0.62 (95% CI 0.59–0.63), p < 0.001; rho = 0.47 (95% CI 0.35–0.51), p < 0.001, respectively). Affective and Sensory subscale scores were positively correlated with each other (rho = 0.48 (95% CI 0.44–0.52), p < 0.001) and linear regression including both subscales as covariates showed significant associations of CAP-Knee scores with each of Affective and Sensory scores (Affective: *B* = 0.65 (95% CI 0.44–0.85), p < 0.001; Sensory: *B* = 0.14 (95% CI 0.06–0.21), p < 0.001).

## Discussion

We show that a Central Mechanisms trait, measured by a psychometrically validated CAP-Knee questionnaire was associated both with sensory and affective components of knee pain^8^^,^^9^. CAP-Knee items were understood well by people with knee pain, aligned to our mechanistic model of central pain augmentation. CAP-Knee scores were strongly associated with worse knee pain, consistent with contributions from central mechanisms to both sensory and affective pain components. CAP-Knee is suitable for use in research measuring the Central Mechanisms trait to develop, target and evaluate treatments that aim to reduce the burden of knee pain by reducing central pain augmentation.

The current study confirms our previous findings in another participant cohort that items addressing each of eight characteristics of neuropathic-like pain, fatigue, cognitive-impact, catastrophizing, anxiety, sleep disturbance, depression, and pain distribution contribute to a single ‘Central Mechanisms’ trait^9^. We show here that the Central Mechanisms trait as measured by CAP-Knee is associated with each of the sensory and affective subscales of the MPQ. This supports an integrative model of knee pain in which central mechanisms contribute both to its severity and to its psychological impact. We have also identified heterogeneous participant interpretations of CAP-Knee items, although interpretations aligned well to our mechanistic model of central pain augmentation. Each of these participant-centred interpretations might be indicative of central pain mechanisms, and each also reflects an area of concern to people with knee pain. These findings highlight the diversity and impact of knee pain within the study population.

Rasch analyses demonstrated that respondents might have difficulty distinguishing between response categories ‘often’ and ‘always’. Scoring transformation that collapsed these responses (each scored 2) resolved disordered thresholds, improved the Rasch properties of the CAP-Knee, and met the Rasch assumption for unidimensionality of the latent trait of Central Mechanisms measured by the CAP-Knee, without otherwise impairing CAP-Knee's psychometric properties. We therefore suggest use of these Rasch transformed scores in future studies to aid longitudinal tracking of the Central Mechanisms trait, or as an outcome measure using parametric analyses. The good measurement properties of CAP-Knee demonstrated here are prerequisite for its use as a mechanistic outcome tool measuring the ability of an intervention to reduce central pain mechanisms.

Our current study is subject to some limitations. Characteristics additional to those eight measured by CAP-Knee, some as yet unidentified, might additionally contribute to central pain mechanisms. Our study might be affected by sources of bias. Study participants were recruited through the IMH&W survey study, which itself recruited from a range of sources in order to optimize the generalizability of our findings. However, different participant samples might have given different results. CAP-Knee was completed following postal receipt by participants, a key advantage if to be used in epidemiological research or surveys.

While previous work demonstrates that the Central Mechanisms trait performs fairly across age, sex and BMI groups, the current work identified DIF by sex for the pain distribution item. This finding reflects previous work which identified women are more likely to report greater widespread pain^57^^,^^58^. The pain distribution item contributed least to the Central Mechanisms trait identified in this study. In both our current and previous study^9^, the distribution item loaded less strongly to the Central Mechanisms trait factor than did other items, suggesting that pain distribution might less reliably indicate central pain mechanisms than do other characteristics. However, based on our earlier data and an extensive literature indicating that widespread pain, as seen in fibromyalgia, is associated with central sensitisation, we recommend retention of the ‘Pain Distribution’ item within CAP-Knee.

Ours is a cross-sectional investigation, and future longitudinal research should determine whether CAP-Knee equals or improves upon outcome prediction by other measures of central pain mechanisms, such as QST^59^. CAP-Knee might also help identify areas of concern for people with knee pain, but does not include items addressing characteristics of additional concern to patients (e.g., swelling or stiffness) that were not linked to central pain mechanisms. CAP-Knee might be useful as an outcome measure to evaluate treatments aiming to reduce central pain mechanisms, but was not designed as a patient-centred outcome measure. Application of CAP-Knee as a stratification tool would require future work to determine clinically relevant cut-off scores, for example, to determine subgroups with knee pain who will have poor pain prognosis, or respond to a drug or psychological intervention designed to modify central pain mechanisms. CAP-Knee would require further validation before use in conditions other than knee pain. Combination of CAP-Knee with other predictors of poor pain prognosis might best identify people most likely to benefit from interventions which target central pain mechanisms^8^.

In conclusion, our findings indicate the heterogeneous nature and impact of knee pain. We describe and validate the CAP-Knee questionnaire, suitable for measuring the Central Mechanisms trait in people with knee pain. We have demonstrated construct validity of the CAP-Knee as a measure of the Central Mechanisms trait, and shown convergent validity of the CAP-Knee with existing sensory and affective measures. Eight self-report items are related to eight discrete characteristics of Neuropathic-like pain, Fatigue, Cognitive-impact, Catastrophising, Anxiety, Sleep disturbance, Depression, and Pain distribution. CAP-Knee is short, easy and inexpensive to administer, either by post or face:face, and was easily understood by people with knee pain. CAP-Knee is suitable for further development as a stratification tool to identify people who might most benefit from new or established treatments designed to reduce augmentation of knee pain by the central nervous system.

## Funding

This work was supported by the 10.13039/501100000837University of Nottingham acting as Sponsor and host institution. This research was co-funded by Pain Centre Versus Arthritis (Centre initiative grant number 20777) and by the 10.13039/501100000272NIHR Nottingham 10.13039/100014461Biomedical Research Centre. The views expressed are those of the author(s) and not necessarily those of the NHS, the NIHR or the Department of Health and Social Care.

## Role of the funding source

Versus Arthritis UK (Centre initiative grant number = 20777), and University of Nottingham as sponsor and host institution. Study sponsors had no role in the design and conduct of the study; collection, management, analysis and interpretation of data; and preparation, review or approval of manuscript.

## Contributions

Kehinde Akin-Akinyosoye, Daniel McWilliams, Roshan das Nair, Eamonn Ferguson and David A Walsh contributed to the conception and design of the interview study. Kehinde Akin-Akinyosoye, Daniel McWilliams, Bonnie Millar, Eamonn Ferguson and David A Walsh contributed to the conception and design of the IMH&W study. Kehinde Akin-Akinyosoye collected the data. Kehinde Ain-Akinyosoye and Richard E James contributed to the coding the interview data. Kehinde Akin-Akinyosoye performed the statistical and qualitative analysis. Kehinde Akin-Akinyosoye, Daniel McWilliams, Roshan das Nair, Eamonn Ferguson and David A Walsh contributed to the data interpretation. All authors critically reviewed and edited the manuscript and approved the final version.

## Declaration of competing interest

Kehinde Akin-Akinyosoye: None declared.

Richard J.E. James: None declared.

Bonnie Millar: None declared.

Daniel F. McWilliams: None declared.

Roshan das Nair: None declared.

Eamonn Ferguson: None declared.

David A. Walsh: None declared.

## References

[1] Felson D.T., Lawrence R.C., Dieppe P.A., Hirsch R., Helmick C.G., Jordan J.M. (2000). Osteoarthritis: new insights. Part 1: the disease and its risk factors. Ann Intern Med.

[2] Wylde V., Beswick A.D., Dennis J., Gooberman-Hill R. (2017). Post-operative patient-related risk factors for chronic pain after total knee replacement: a systematic review. BMJ open.

[3] Arendt-Nielsen L., Egsgaard L.L., Petersen K.K., Eskehave T.N., Graven-Nielsen T., Hoeck H.C. (2015). A mechanism-based pain sensitivity index to characterize knee osteoarthritis patients with different disease stages and pain levels. Eur J Pain.

[4] Wylde V., Palmer S., Learmonth I.D., Dieppe P. (2013). The association between pre-operative pain sensitisation and chronic pain after knee replacement: an exploratory study. Osteoarthritis Cartilage.

[5] Woolf C.J. (2011). Central sensitization: implications for the diagnosis and treatment of pain. Pain.

[6] Enteshari-Moghaddam A., Azami A., Isazadehfar K., Mohebbi H., Habibzadeh A., Jahanpanah P. (2019). Efficacy of duloxetine and gabapentin in pain reduction in patients with knee osteoarthritis. Clin Rheumatol.

[7] das Nair R., Mhizha-Murira J.R., Anderson P., Carpenter H., Clarke S., Groves S. (2018). Home-based pre-surgical psychological intervention for knee osteoarthritis (HAPPiKNEES): a feasibility randomized controlled trial. Clin Rehabil.

[8] Akin-Akinyosoye K., Sarmanova A., Fernandes G.S., Frowd N., Swaithes L., Stocks J. (2020). Baseline self-report ‘central mechanisms’ trait predicts persistent knee pain in the Knee Pain in the Community (KPIC) cohort. Osteoarthritis Cartilage.

[9] Akin-Akinyosoye K., Frowd N., Marshall L., Stocks J., Fernandes G.S., Valdes A. (2018). Traits associated with central pain augmentation in the Knee Pain in the Community (KPIC) cohort. Pain.

[10] Ali A., Lindstrand A., Sundberg M., Flivik G. (2017). Preoperative anxiety and depression correlate with dissatisfaction after total knee arthroplasty: a prospective longitudinal cohort study of 186 patients, with 4-year follow-up. J Arthroplasty.

[11] Campbell C.M., Buenaver L.F., Finan P., Bounds S.C., Redding M., McCauley L. (2015). Sleep, pain catastrophizing, and central sensitization in knee osteoarthritis patients with and without insomnia. Arthritis Care Res.

[12] Lluch E., Nijs J., Courtney C.A., Rebbeck T., Wylde V., Baert I. (2018 Nov 6). Clinical descriptors for the recognition of central sensitization pain in patients with knee osteoarthritis. Disabil Rehabil.

[13] Brown D., Mulvey M., Cordingley L., Rashid A., Horan M., Pendleton N. (2016). The relationship between psychological distress and multiple tender points across the adult lifespan. Arch Gerontol Geriatr.

[14] Nishigami T., Tanaka K., Mibu A., Manfuku M., Yono S., Tanabe A. (2018). Development and psychometric properties of short form of central sensitization inventory in participants with musculoskeletal pain: a cross-sectional study. PloS One.

[15] van Bemmel P.F., Voshaar M.A., Ten Klooster P.M., Vonkeman H.E., van de Laar M.A. (2019). Development and preliminary evaluation of a short self-report measure of generalized pain hypersensitivity. J Pain Res.

[16] Gwilym S.E., Keltner J.R., Warnaby C.E., Carr A.J., Chizh B., Chessell I. (2009). Psychophysical and functional imaging evidence supporting the presence of central sensitization in a cohort of osteoarthritis patients. Arthritis Care Res: Official Journal of the American College of Rheumatology..

[17] Hochman J.R., Davis A.M., Elkayam J., Gagliese L., Hawker G.A. (2013). Neuropathic pain symptoms on the modified painDETECT correlate with signs of central sensitization in knee osteoarthritis. Osteoarthritis Cartilage.

[18] Lluch E., Torres R., Nijs J., Van Oosterwijck J. (2014). Evidence for central sensitization in patients with osteoarthritis pain: a systematic literature review. Eur J Pain.

[19] Moreton B.J., Tew V., das Nair R., Wheeler M., Walsh D.A., Lincoln N.B. (2015). Pain phenotype in patients with knee osteoarthritis: classification and measurement properties of painDETECT and self-report Leeds Assessment of Neuropathic Symptoms and Signs Scale in a cross-sectional study. Arthritis Care Res.

[20] Blair J., Conrad F.G. (2011). Sample size for cognitive interview pretesting. Publ Opin Q.

[21] Perneger T.V., Courvoisier D.S., Hudelson P.M., Gayet-Ageron A. (2015). Sample size for pre-tests of questionnaires. Qual Life Res.

[22] Willis G.B., Artino A.R. (2013). What do our respondents think we're asking? Using cognitive interviewing to improve medical education surveys. Journal of graduate medical education.

[23] Millar B., McWilliams D.F., Abhishek A., Akin-Akinyosoye K., Auer D.P., Chapman V. (2020 Dec). Investigating musculoskeletal health and wellbeing; a cohort study protocol. BMC Muscoskel Disord.

[24] Chen W.H., Lenderking W., Jin Y., Wyrwich K.W., Gelhorn H., Revicki D.A. (2014). Is Rasch model analysis applicable in small sample size pilot studies for assessing item characteristics? An example using PROMIS pain behavior item bank data. Qual Life Res.

[25] Paiva C.E., Barroso E.M., Carneseca E.C., de Pádua Souza C., Dos Santos F.T., López R.V. (2014). A critical analysis of test-retest reliability in instrument validation studies of cancer patients under palliative care: a systematic review. BMC Med Res Methodol.

[26] Melzack R., Wall P.D. (1996). The Challenge of Pain.

[27] Status W.P. (1995). The use and interpretation of anthropometry. WHO Tech Rep Ser.

[28] Fernandes G.S., Sarmanova A., Warner S., Harvey H., Akin-Akinyosoye K., Richardson H. (2017). Knee pain and related health in the community study (KPIC): a cohort study protocol. BMC Muscoskel Disord.

[29] Hochman J.R., Gagliese L., Davis A.M., Hawker G.A. (2011). Neuropathic pain symptoms in a community knee OA cohort. Osteoarthritis Cartilage.

[30] Ware J.E., Kosinski M., Keller S.D. (1996). A 12-Item Short-Form Health Survey: construction of scales and preliminary tests of reliability and validity. Med Care.

[31] Ferguson E., Daniel E. (1995). The Illness Attitudes Scale (IAS): a psychometric evaluation on a non-clinical population. Pers Indiv Differ.

[32] Sullivan M.B.S.R., Pivik J. (1995). The pain catastrophizing scale: development and validation. Psychol Assess.

[33] Zigmond A.S., Snaith R.P. (1983). The hospital anxiety and depression scale. Acta Psychiatr Scand.

[34] Lacey R.J., Lewis M., Jordan K., Jinks C., Sim J. (2005). Interrater reliability of scoring of pain drawings in a self-report health survey. Spine.

[35] Altman R., Asch E., Bloch D., Bole G., Borenstein D., Brandt K. (1986). Development of criteria for the classification and reporting of osteoarthritis: classification of osteoarthritis of the knee. Arthritis Rheum: Official Journal of the American College of Rheumatology..

[36] Beatty P.C., Willis G.B. (2007). Research synthesis: the practice of cognitive interviewing. Publ Opin Q.

[37] Tourangeau R. (1984).

[38] QSR International (2018). NVIVO 12 Plus.

[39] Lewis S. (2015). Qualitative inquiry and research design: choosing among five approaches. Health Promot Pract.

[40] Hsieh H.F., Shannon S.E. (2005). Three approaches to qualitative content analysis. Qual Health Res.

[41] Efremova M., Panyusheva T., Schmidt P., Zercher F. (2017). Mixed methods in value research: an analysis of the validity of the Russian version of the schwartz value survey (SVS) using cognitive interviews, multidimensional scaling (MDS), and confirmatory factor Analysis (CFA). ASK. Research and Methods.

[42] Braun V., Clarke V. (2006). Using thematic analysis in psychology. Qual Res Psychol.

[43] DeSantis L., Ugarriza D.N. (2000). The concept of theme as used in qualitative nursing research. West J Nurs Res.

[44] Jarrett N.J., Payne S.A., Wiles R.A. (1999). Terminally ill patients' and lay-carers' perceptions and experiences of community-based services. J Adv Nurs.

[45] Moreton B.J., Wheeler M., Walsh D.A., Lincoln N.B. (2012). Rasch analysis of the intermittent and constant osteoarthritis pain (ICOAP) scale. Osteoarthritis Cartilage.

[46] Computing R.R. (2013). A Language and Environment for Statistical Computing.

[47] Ramp M., Khan F., Misajon R.A., Pallant J.F. (2009). Rasch analysis of the multiple sclerosis impact scale (MSIS-29). Health Qual Life Outcome.

[48] Pallant J.F., Tennant A. (2007). An introduction to the Rasch measurement model: an example using the Hospital Anxiety and Depression Scale (HADS). Br J Clin Psychol.

[49] Smith A.B., Rush R., Fallowfield L.J., Velikova G., Sharpe M. (2008). Rasch fit statistics and sample size considerations for polytomous data. BMC Med Res Methodol.

[50] Wright B.D., Stone M.H. (1979). Best Test Design.

[51] Romanoski J., Douglas G. (2002). Test scores, measurement, and the use of analysis of variance: an historical overview. J Appl Meas.

[52] Muthén M. (2012). MPLUS:.

[53] StataCorp L.P. (2015). StataCorp. Stata Statistical Software: Release.

[54] Streiner D.L., Norman G.R., Cairney J. (2015). Health Measurement Scales: A Practical Guide to Their Development and Use.

[55] Koo T.K., Li M.Y. (2016). A guideline of selecting and reporting intraclass correlation coefficients for reliability research. Journal of chiropractic medicine.

[56] Arendt-Nielsen L., Morlion B., Perrot S., Dahan A., Dickenson A., Kress H.G. (2018). Assessment and manifestation of central sensitisation across different chronic pain conditions. Eur J Pain.

[57] Leveille S.G., Zhang Y., McMullen W., Kelly-Hayes M., Felson D.T. (2005). Sex differences in musculoskeletal pain in older adults. Pain.

[58] Bartley E.J., King C.D., Sibille K.T., Cruz-Almeida Y., Riley J.L., Glover T.L. (2016). Enhanced pain sensitivity among individuals with symptomatic knee osteoarthritis: potential sex differences in central sensitization. Arthritis Care Res.

[59] Georgopoulos V., Akin-Akinyosoye K., Zhang W., McWilliams D.F., Hendrick P., Walsh D.A. (2019). Quantitative Sensory Testing (QST) and predicting outcomes for musculoskeletal pain, disability and negative affect: a systematic review and meta-analysis. Pain.

