# Supplementary material for: The Central Aspects of Pain in the Knee (CAP-Knee) questionnaire; a mixed-methods study of a self-report instrument for assessing central mechanisms in people with knee pain
Source: Osteoarthritis Cartilage. 2021 Jun;29(6):802–14. doi: 10.1016/j.joca.2021.02.562 (PMC8177001; doi:10.1016/j.joca.2021.02.562)
Supplement: Multimedia component 1 [file mmc1.docx]

# SUPPLEMENT 1. INTERVIEW GUIDE FOR COGNITIVE INTERVIEW

Introduction

*“Hello, my name is Kehinde Akin-Akinyosoye. I am a PhD student with the Arthritis Research UK Pain Centre at Nottingham University. Thank you for agreeing to participate in this interview. Your feedback will help us learn how people with knee pain interpret the questions within this questionnaire. The purpose for this interview is to find out your understanding of each question, particularly, how you come to understand these questions.”*

*“I will be recording the interview. Do I have your permission to record the interview?”*

- If yes, start the recording equipment and read the consent form to the interviewee.
- If no, terminate the interview. Offer participant inclusion to the reliability study to the participant.

*“Before we start, I just have a few things to tell you”.*

*“This interview will only take 30 minutes depending on how much you would like to say in answer to each question”*

*“If you would like to withdraw from the study and stop the interview at any time, then you can do so without giving a reason. If you would like to have a break during the interview, you are also free to do so. I will pause the audio recording at that point until we begin the interview again.”*

*“For this interview, I will hand you a blank version of the questionnaire. The questionnaire contains 8 questions which we will be discussing today. I will like you to complete this questionnaire, and I will then ask you about how you understood and responded to each question.*

*“Do you have any questions before we begin?”*

- Answer any questions
- Hand over blank questionnaire to participant

“Please remember that there are no right or wrong answers, and what you say will not hurt my feelings. Feel free to say anything that you are thinking”

“Are you happy to go ahead with the interview?

- If yes, begin interview: “*Okay, let’s begin with the first question*”
- If no, ask: *“Are there any concerns that you would like me to address?”*

**Question 1: Cold or heat touching my knee was painful** (interviews 1-17) or **Cold or heat (e.g. bath water) on my knee was painful** (interviews 18-22)

Probe:

- *“Can you tell me in your own words, what the statement means to you?”*

If participant shows difficulty responding: *“Can you repeat the question I just asked in your own words?”*

- *“What were you thinking of when you answered the statement?”*

If participant shows difficulty responding: *“How did you go about deciding on which answer to pick?”*

- *“How sure are you of your answer?”*

**Question 2: I generally felt tired**

Probe:

- *“Can you tell me in your own words, what the statement means to you?”*

If participant shows difficulty responding: *“Can you repeat the question I just asked in your own words?”*

- *“What were you thinking of when you answered the statement?”*

If participant shows difficulty responding: *“How did you go about deciding on which answer to pick?”*

- *“How sure are you of your answer?”*

**Question 3: My knee pain stopped me concentrating on what I was doing**

Probe:

- *“Can you tell me in your own words, what the statement means to you?”*

If participant shows difficulty responding: *“Can you repeat the question I just asked in your own words?”*

- *“What were you thinking of when you answered the statement?”*

If participant shows difficulty responding: *“How did you go about deciding on which answer to pick?”*

- *“How sure are you of your answer?”*

**Question 4: I kept thinking about how much my knee hurts**

Probe:

- *“Can you tell me in your own words, what the statement means to you?”*

If participant shows difficulty responding: *“Can you repeat the question I just asked in your own words?”*

- *“What were you thinking of when you answered the statement?”*

If participant shows difficulty responding: *“How did you go about deciding on which answer to pick?”*

- *“How sure are you of your answer?”*

**Question 5: In general, I got sudden feelings of panic**

Probe:

- *“Can you tell me in your own words, what the statement means to you?”*

If participant shows difficulty responding: *“Can you repeat the question I just asked in your own words?”*

- *“What were you thinking of when you answered the statement?”*

If participant shows difficulty responding: *“How did you go about deciding on which answer to pick?”*

- *“How sure are you of your answer?”*

**Question 6: My knee pain affected my sleep**

Probe:

- *“Can you tell me in your own words, what the statement means to you?”*

If participant shows difficulty responding: *“Can you repeat the question I just asked in your own words?”*

- *“What were you thinking of when you answered the statement?”*

If participant shows difficulty responding: *“How did you go about deciding on which answer to pick?”*

- *“How sure are you of your answer?”*

**Question 7: I generally still enjoyed the things I used to enjoy**

Probe:

- *“Can you tell me in your own words, what the statement means to you?”*

If participant shows difficulty responding: *“Can you repeat the question I just asked in your own words?”*

- *““What were you thinking of when you answered the statement?”*

If participant shows difficulty responding: *“How did you go about deciding on which answer to pick?”*

- *“How sure are you of your answer?”*

**Question 8: This final question is about pain you may have had in any part of your body. Please shade in the diagram below, to indicate where you have suffered any pain for most days in the last 4 WEEKS. By pain we also mean aching and/or discomfort. Please do not include pain due to feverish illness such as flu**

Probe:

- *“Can you tell me in your own words, what the statement means to you?”*

If participant shows difficulty responding: *“Can you repeat the question I just asked in your own words?”*

- *“What were you thinking of when you were shading in the picture?”*

If participant shows difficulty responding: *“How did you go about deciding on how to shade in the picture?”*

- *“Can you tell me how long have you felt pain in the areas that you shaded?”*
- *“How sure are you of your answer?”*

**Closing**

*“That was my final question. I will now switch of the audio recording equipment.”*

Switch off recording equipment

*“Thank you for taking time to answer these questions and for your participation in the study. Please feel free to share any other comments that you haven’t shared to this point”*

- Pause to allow the interviewee to share additional comments.

*“If there are questions or concerns, please contact me on [telephone number].”*

# SUPPLEMENT 2. ITEM CODING SCHEME

Participant *comprehension* of each CAP-Knee item was determined by coding within themes that were identified through interview by reference to the researcher’s intended meaning of that item (listed below). Comprehension was coded as completely aligned, partially aligned or completely non-aligned. *Retrieval difficulty* was coded as none, partial or complete, based on the extent to which participants directly addressed the intended meaning of the item, or instead referred to other aspects of the item before concluding their response to its intended meaning. For example, responses to item 1 were coded as partial retrieval difficulty if participants recalled event(s) not related as well as those that were related to hot or cold stimuli causing or not causing pain to the affected knee. *Judgement* was coded as `certain’ or `uncertain’ initial response according to whether the participant indicated during interview that they were certain or unsure whether they agreed with the response that they had provided on the hard copy CAP-Knee questionnaire prior to commencing the interview, and whether the questionnaire response matched their responses during the interview. For example, judgement was coded as `uncertain’ for item 1 for a participant who reported during interview that heat or cold on their knee sometimes produced pain, but who had responded “never” on the questionnaire, or if the participant was unable to decide on a response for the question. *Response consistency* was coded as `Consistent’ (only one response category checked per item), or `Inconsistent’ (no or >1 response category checked).

Codes were developed and validated by discussion between 2 researchers (KAA and RJEJ) until consensus was reached, using transcripts from 7 participants.

**Item intended meanings**

**Neuropathic-like pain:**  ‘Cold or heat touching my knee was painful’ (interviews 1-17) or `Cold or heat (e.g. bath water) on my knee was painful’ (interviews 18-22)

Hypersensitivity to heat or cold applied to the knee, where stimulus intensity is lower than would normally be required to cause pain or damage, indicative of thermal allodynia (Phillips 2017) . Application of a cold or hot stimulus that would normally not cause pain when in physical contact with unaffected areas, causes pain to the participant’s knee.

**Fatigue:**  ‘I generally felt tired’

The enduring, subjective sensation of generalized tiredness or exhaustion, which may or may not be related to knee pain. Fatigue, or feeling tired is typically also described as feeling “rundown” or “knackered”, physically and/or mentally.

**Cognitive-impact:** ‘My knee pain stopped me concentrating on what I was doing’

Concentration is an attentional process that involves the ability to focus on the task at hand while ignoring distractions (Moran 2012). Knee pain might interfere with, interrupt, distract, or prevent someone from concentrating on something they were previously focused on.

**Catastrophizing:** ‘I kept thinking about how much my knee hurts’

This item investigates the focused attention on knee pain, on its possible causes and consequences, as opposed to its solutions, indicative of ruminative thoughts (Hilt 2007) . This item seeks to identify the frequency of ruminative thoughts about having knee pain, and the knee pain experience.

**Anxiety:** ‘In general, I got sudden feelings of panic’

Panic is an extreme form of fear and anxiety, and is characterized by physical sensations, such as a racing heart, shortness of breath that lasts for a short period of time. Panic is a fear of unfamiliar situations, triggered by frightening thoughts, images, and sensations (Busch 1993) . This item seeks to identify whether the respondent has had feelings of panic, nervousness or unease, which might or might not be related to knee pain.

**Sleep:** ‘My knee pain affected my sleep’

This item investigates the frequency of knee pain disrupting sleep (nocturnal or daytime), including poor quality of sleep, insufficient or too much inefficient sleep, or interrupting sleep cycle.

**Depression:** ‘I generally still enjoyed the things I used to enjoy’

This item addresses a loss of interest or pleasure (Bartolomucci 2009) . Enjoyment is the feeling of pleasure, satisfaction or happiness that you have when you do or experience something that you like.

**Pain distribution:** ‘This final question is about pain you may have had in any part of your body. Please shade in the diagram below, to indicate where you have suffered any pain for most days in the last 4 WEEKS. By pain we also mean aching and/or discomfort. Please do not include pain due to feverish illness such as flu’

This item investigates location of pain, ache or discomfort across anatomical sites.

**References**

Bartolomucci A, Leopardi R. Stress and depression: preclinical research and clinical implications. PloS one. 2009;4(1):e4265.

Busch FN, Shapiro T. A psychodynamic model of panic disorder. Am J Psychiatry. 1993;150(6):859-66.

Hilt LM, Sander LC, Nolen-Hoeksema S, Simen AA. The BDNF Val66Met polymorphism predicts rumination and depression differently in young adolescent girls and their mothers. Neuroscience letters. 2007;429(1):12-6

Moran A. Concentration: Attention and performance. The Oxford handbook of sport and performance psychology Oxford University Press, Northampton. 2012:117-30

Phillips JRA, Hopwood B, Stroud R, Dieppe PA, Toms AD. The characterisation of unexplained pain after knee replacement. British journal of pain. 2017;11(4):203-9.

# SUPPLEMENT 3. CAP-KNEE RASCH-TRANSFORMED SCORING

*Rasch-transformed scoring algorithm for CAP-Knee*

Items 1-7

Never = 0

Sometimes = 1

Often =2

Always =2

Item 8: Pain distribution.

Manikin:

No shaded regions below waist = 0.

Only 1 knee shaded and no other regions below the waist = 0.

1 knee plus any additional shaded region below the waist = 2.

Both knees shaded = 2.

**CAP-Knee score**

**Sum of items 1, 2, 3, 4, 5, 6, 7, 8**

**Range 0-16**
