# Supplementary figures and images for: The Central Aspects of Pain in the Knee (CAP-Knee) questionnaire; a mixed-methods study of a self-report instrument for assessing central mechanisms in people with knee pain
Source: Osteoarthritis Cartilage. 2021 Jun;29(6):802–14. doi: 10.1016/j.joca.2021.02.562 (PMC8177001; doi:10.1016/j.joca.2021.02.562)

# Supplementary Figure 1: Original Version of the CAP-Knee Scale


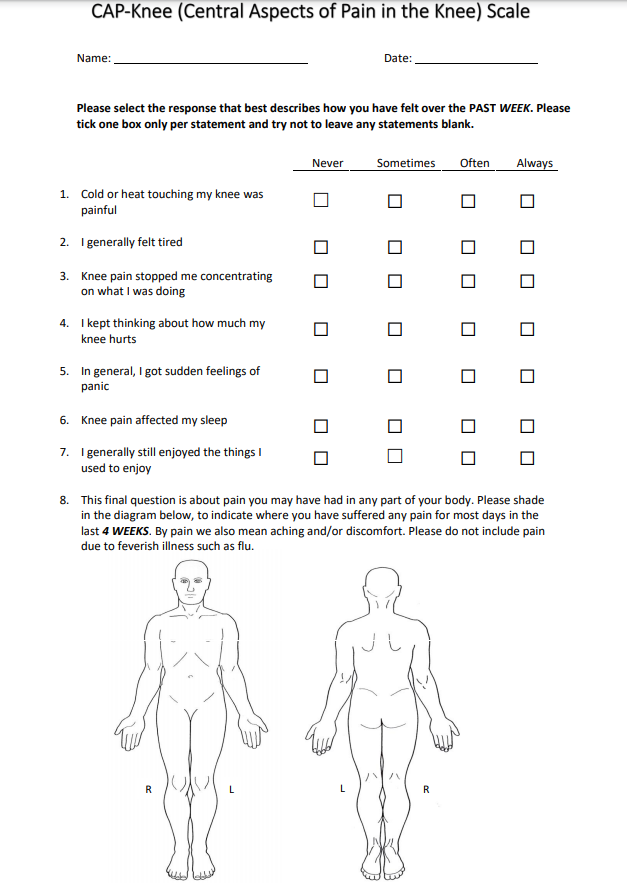

Supplement: Multimedia component 2 [file mmc2.docx]
